# Shakespeare's Medical Knowledge

**Published:** 1916-05-13

**Authors:** 


### SHAKESPEARE'S MEDICAL KNOWLEDGE.

### "One of our Greatest Masters of Medicine."

The exordium of Sir St. Clair Thomson's oration on "Shakespeare and Medicine" before the London Medical Society on May 1, to which we drew attention last week, contained the following passage: "I have made no investigations, and have no new readings to submit. If my oration is . . . made up of quotations . . . yet I remember that Emerson said 'it is as difficult to quote the thoughts of others as it is to invent.'" This is a just piece of criticism, but the speaker might have added, with equal justice, that a string of quotations is as dubious a way of presenting a great man's thoughts as could be hit upon, especially when the desire to emphasise an author's bias for a particular side or knowledge of a particular subject is the principal object in view. The mind of the poet is a synthetic mind, delighting in generalisations and in the detection of analogies between apparently remote objects. As the poet is also exceedingly curious of facts, and ever on the watch for the peculiar idioms of farm and market, court and kitchen, delighting especially, for their raciness, in the technical terms of every trade and profession, one mark of the born poet is the use of very varied similes and metaphors, which have been noted down wherever and whenever he happened to hear them. But in the very act of so employing them the poet will be thinking not of the trade from which this or that happy illustration has been drawn, but of the idea whose expression can be most aptly pointed by the vivid language of the workshop, the barn, the office, or the laboratory. As Dr. Johnson well says of Shakespeare's dialogue, "it seems to have been gleaned by diligent selection out of common conversation and common occurrences."

Once upon a time W. B. Yeats and George Moore were speaking of dialects. Yeats said, "By-paths are always beautiful: it is the broad road of the journalist that is ugly." In recording this Moore exclaimed, just as Shakespeare would have done, "Such picturesqueness of speech always enchants me"; but then he is a man of letters too. Could anything better show the tendency of a poet's mind, and the motive behind the traditional manner of his writing?

It is therefore a great temptation for any professional man, when coming across this or that technical phrase drawn from his own line of business in the writings of a great poet, to imagine over-fondly that the author must have had some particular acquaintance with his own profession. The bare fact that, as we noted last week, books have been written to connect Shakespeare with the printing, the lawyer's, even the soldier's trade is enough to prove that the temptation "to claim him" is stronger than the common-sense of most of his professed admirers.

With the caution implied in the preceding paragraph, we are free to examine, with some chance of getting value for our pains, the lengthy series of quotations, carefully grouped under their respective headings, which the extensive industry of Sir St. Clair Thomson has compiled.

As we are reminded in the useful bibliographical note appended to this oration, the subject of Shakespeare and Medicine has a pigeon-hole of its own in the commentators' library. Indeed, without enjoying or digesting, or even reading, one of his plays, from Arthur W. Meyer's *The Physician and Surgeon in Shakespeare*; from James F. West's *William Shakespeare from a Surgeon's Point of View*; from Albert C. Getchell's *The Medical Knowledge of Shakespeare*, and P. S. Donellan's *Medical Allusions in Shakespeare's Plays*—without digesting or enjoying one of his plays, it would be possible from such articles as these to display a formidable list of medical allusions, more or less apt. The first step is to give some sort of perspective to this haphazard agglomeration, as Sir St. Clair has begun to do by grouping his quotations under various headings.

His main classification is as follows:—

Shakespeare as a medical seer; the physicians in the plays; Shakespeare's general medical knowledge; the herbs and drugs mentioned by him, and so on. To these are added allusions, or what are surmised to be allusions, to the dressing of wounds, to blood-letting; to the circulation of the blood; to his list of the signs of death; to air and climate; to syphilis; and, lastly, to his sympathies and his remarks on the psychological value of hope and mirth, his praise of sleep, his pictures of old age and of death, both normal and sudden.

In studying these examples of his interest in medical matters, we shall not go pedantically astray, or twist a general application unduly to illustrate some medical allusion that may perhaps be associated with it, if we remember his best editor's warning that "he that will understand Shakespeare must not be content to study him in the closet; he must look for his meaning sometimes among the sports of the field, and sometimes among the manufactures of the shop." Moving among all and sundry, Shakespeare was a snapper-up of unconsidered trifles, with, like Hamlet, his tablets always ready to set down whatever good thing or fine cadence happened to be dropped in the unconscious, and therefore idiomatic, talk of those about him. Among these must have been physicians, and it is, one thinks, rather among such as he happened to meet in friends' houses, at stage-doors, about the Court, and in the Tube (so to speak) that we should look for supplying his medical observations than to the more formal circumstance that, towards the end of his life, his daughter married Dr. Hall. A man and his son-in-law rarely learn much from one another.

Many of his medical aphorisms, like "With eager feeding food doth choke the feeder," are, of course, instances of general observation merely. Shakespeare's aptitude was not so much for book-learning as for attention to what was being said or done under his nose: a much rarer gift. It is fair to add that Sir St. Clair Thomson does not quote this line as an example of Shakespeare's knowledge of dietetics and digestion; but he does overstate his case. Any observant person might have said, "Unquiet meals make ill digestions," and surely it is far-fetched to suggest that Hamlet may be anticipating "our twentieth-century studies of cardiac rhythm" when he says to his mother:

> My pulse, as yours, doth temperately keep time,
> And makes as healthful music.

Or again, when the Ghost in *Hamlet* describes the action of the poison, which was poured in the porches of his ears, as one

> Whose effect
> Holds such an enmity with blood of man
> That swift as quicksilver it courses through
> The natural gates and alleys of the body;
> And with a sudden vigour it doth posset
> And curd, like eager droppings into milk,
> The thin and wholesome blood.

Is not Sir St. Clair Thomson straining too far when he says, "It is almost uncanny to read these lines, conveying, as they do, at first glance, a comprehension of the circulation of the blood"? "For" (he adds) "by a strange coincidence it was only in the year 1616, and in the month of April, and in the very week preceding Shakespeare's death, that William Harvey . . . first enunciated his memorable discovery." These lines have always seemed to the present writer a capital instance of the way in which a man of letters will disguise his ignorance of a technical process by a generalised but pleasant imagery, whose object and effect, when successful, is to supply by a swelling figure in several lines what more exact comprehension would be able to condense into a single line or phrase.

Let us turn, however, to one or two of such precise instances as are available in the more agreeable task of showing what a fund of observation Shakespeare had.

Take this true rule, perhaps the best of all, and one which is still so often disregarded by nurses:—

> I come to bring him sleep. 'Tis such as you—
> That creep like shadows by him, and do sigh
> At each his needless heavings—such as you
> Nourish the cause of his awaking.

How irresistibly doctor and nurse are reminded by this of Florence Nightingale's admirable remarks on noise: Walking on tip-toe, doing anything in the room very slowly, are injurious; for the patient's attention, as in whispered conversations, is involuntarily strained to hear. A firm, light, quick step, a steady, quick hand are what you want.

The examples given by Sir St. Clair of Shakespeare's familiarity with purely professional matters include his specific mention of *tremor cordis*, *hysterica passio*, and the *pia mater;* but in quoting from *King John*

> The life of all his blood
> Is touched corruptibly; and his pure brain
> (Which some suppose the soul's frail dwelling-house), etc., the writer might have added that, by parity of reasoning with the other examples quoted, this may perhaps be taken as referring to the pineal body.

But when we have come to an end of all examples, and the curious should certainly study them at full length in this elaborate oration, we must fall back, in the speaker's own words, on Shakespeare's "profound knowledge of human life and human nature" to explain "why he is, and must always be, one of our greatest masters in medicine." Robert Browning was another of his kidney, with the same love of technical terms and the same store of out-of-the-way knowledge. The musician, the doctor, the lawyer, the ornithologist can find in both the same delight in the details of their crafts, and the same observation and sympathy which are the mark of the born poet. But behind all this an ear for word music is the real key to both of them.

